# Risk Factors Associated with Spinal Cord Stimulation Failure in Patients with Chronic Pain: A Single-Center Retrospective Cohort Study

**DOI:** 10.3390/jcm15197681

**Published:** 2026-10-04

**Authors:** Ahmed Osailan, Ahmed Najjar, Marie-Pierre Fournier-Gosselin, Francois Fugere

**Affiliations:** 1 Department of General and Specialized Surgery, College of Medicine, Taibah University, Madinah 42353, Saudi Arabia; aosailan@taibahu.edu.sa; 2 Department of Surgery, King Faisal Specialist Hospital and Research Center, Madinah 42523, Saudi Arabia; 3 Division of Neurosurgery, Department of Surgery, Centre Hospitalier de l’Université de Montréal (CHUM), Université de Montréal, Montreal, QC H2X 3E4, Canada; marie-pierre.fournier-gosselin@umontreal.ca; 4 Department of Anaesthesiology and Pain Medicine, Chronic Pain Clinic, Centre Hospitalier de l’Université de Montréal (CHUM), Montreal, QC H2X 3E4, Canada; francois.fugere.med@ssss.gouv.qc.ca

**Keywords:** chronic pain, spinal cord stimulation, treatment failure, neuromodulation, paresthesia, opioid exposure

## Abstract

**Background/Objectives:** Spinal cord stimulation (SCS) can provide long-term relief for selected patients with refractory chronic pain, but treatment failure may occur without device explantation. This study examined patient-, clinical-, and device-related factors associated with SCS failure after permanent implantation. **Methods:** This retrospective, single-center cohort study included 108 adults who underwent permanent SCS implantation at the Centre Hospitalier de l’Université de Montréal between 2007 and 2022. Failure was defined as completed or planned explantation, inadequate pain relief accompanied by a desire or plan for removal, or documented device non-use. Associations were evaluated using univariable categorical tests. Paired pain intensity and morphine milligram equivalent (MME) doses were compared using Wilcoxon signed-rank tests. **Results:** At the most recent documented assessment, composite SCS failure occurred in 40/108 patients (37.0%), and completed explantation occurred in 25/108 (23.1%); these proportions were not adjusted for unequal follow-up duration. The leading reasons for explantation were loss of efficacy (36.0%), paresthesia or dysesthesia (28.0%), and patient request (24.0%). In exploratory univariable analysis, smoking was associated with documented failure (RR = 1.70, 95% CI 1.05–2.75; *p* = 0.032). Failure differed across comorbidity-count categories (Cramér’s V = 0.34, 95% CI 0.00–0.48; *p* = 0.019), although the pattern was nonmonotonic. Neither association remained statistically significant after Benjamini–Hochberg correction across 26 comparisons (both *q* = 0.416). Sex, psychiatric history, pain duration, implantation approach, generator type, and generator location were not significantly associated with failure. The median within-patient reduction in pain intensity was 2.50 points (IQR, 1.00–5.00; *p* < 0.001), whereas the median paired MME change was 0 MME/day (IQR, −60 to 40; *p* = 0.649). **Conclusions:** A composite outcome identified a higher prevalence of clinically unsuccessful SCS treatment than completed explantation alone. Smoking showed an unadjusted association with documented failure; however, this finding may reflect confounding or chance arising from multiple comparisons and should be considered hypothesis-generating. Pain intensity improved without a corresponding significant reduction in opioid exposure. These findings require confirmation through prospective, prespecified multivariable and time-to-event analyses.

## 1. Introduction

Chronic neuropathic pain is a major clinical and public health problem that can substantially impair physical functioning, psychological well-being, social participation, and quality of life. Spinal cord stimulation (SCS) is an established neuromodulation therapy for selected patients whose pain remains refractory to conservative treatments. The therapy involves placing electrodes in the epidural space and delivering electrical impulses through an implantable pulse generator. Although the analgesic effect of SCS was historically explained by the gate control theory of pain, current evidence suggests that it involves multiple spinal and supraspinal mechanisms that modulate nociceptive processing [1,2].

Approximately 50,000 SCS systems are implanted worldwide each year, and their use continues to expand with advances in device technology and stimulation paradigms [2]. Candidates for permanent implantation typically undergo multidisciplinary assessment after pharmacological treatment, physical rehabilitation, and appropriate interventional procedures fail to provide adequate relief. Psychological and psychosocial assessments are also recommended to identify active psychiatric, behavioral, or social factors that may adversely affect treatment adherence and long-term outcomes [3,4]. Trial stimulation is commonly used as a pragmatic screening step before permanent implantation. However, a successful trial does not necessarily predict durable benefit, and evidence supporting individual psychological or clinical predictors remains heterogeneous [3,4].

SCS is used to treat several chronic neuropathic pain conditions, including persistent spinal pain syndrome after surgery—historically termed failed back surgery syndrome—complex regional pain syndrome, refractory low-back and lower-extremity pain, and selected forms of peripheral neuropathic pain [1,3,4,5,6]. In appropriately selected patients, SCS can reduce pain and improve function and quality of life. Earlier consideration of SCS has been associated with better outcomes in some observational studies, although findings on optimal implantation timing remain inconsistent [4,5]. Despite these potential benefits, therapeutic response is not universal and may diminish over time. Device revision, reoperation, and explantation remain clinically and economically important outcomes, with reported incidence varying considerably by treated population, device type, indication, follow-up duration, and outcome definition [7,8,9].

Defining SCS failure is challenging because unsuccessful therapy is not a single clinical event. Failure may involve inadequate initial analgesia, loss of efficacy after an initial response, intolerable paresthesia or dysesthesia, hardware malfunction, lead migration, infection, device non-use, or patient-requested discontinuation. Many previous studies have used explantation as the primary marker of failure because it is an objective, readily identifiable outcome [7,8,9,10]. Nevertheless, explantation alone may underestimate clinically unsuccessful treatment because some patients retain an implanted device despite inadequate pain relief or discontinue its use without removal. Conversely, not every explantation reflects loss of therapeutic efficacy; removal may be required for infection, hardware complications, magnetic resonance imaging requirements, another surgical procedure, or patient preference [8,9,10]. A broader composite outcome may therefore better reflect real-world treatment failure, provided that its components are explicitly defined and, where possible, analyzed separately.

Potential predictors of unsuccessful SCS therapy span patient-, disease-, provider-, procedure-, and device-level domains. Patient-level factors investigated in previous studies include age, biological sex, smoking, psychiatric comorbidity, coping characteristics, comorbidity burden, preimplantation opioid exposure, pain duration, and the underlying pain diagnosis [4,8,11,12,13,14]. However, findings have frequently diverged. Depression, anxiety, and other psychological characteristics have been associated with poorer outcomes in some cohorts but not in others, supporting comprehensive individualized assessment rather than reliance on a single psychological criterion [3,4]. Similarly, higher preimplantation opioid exposure, longer pain duration, and biological sex have shown inconsistent associations across populations and indications [4,12,13,14]. These discrepancies may reflect differences in patient selection, sample size, outcome definitions, follow-up duration, and statistical adjustment for confounding.

Technical and treatment-related factors may also influence outcomes. Lead type and position, implantation technique, stimulation waveform, device programming, provider experience, infection, lead migration, and hardware malfunction may affect revision, reoperation, or explantation risk [10,15,16]. Newer stimulation paradigms, including high-frequency, burst, and other paresthesia-free approaches, may preserve or restore analgesia in some patients who lose benefit from conventional stimulation, but the evidence remains heterogeneous and does not eliminate the possibility of long-term failure [16]. Moreover, devices, imaging compatibility, implantation practices, and selection criteria have changed substantially over time. Calendar year and follow-up duration should therefore be considered when interpreting long-term outcomes. Because patients with older implants have had more opportunities to experience failure, analyses based solely on a binary outcome may be affected by unequal observation time; differences in follow-up duration must therefore be considered when interpreting long-term failure estimates [8,9].

The primary objective of this retrospective single-center cohort study was to examine patient-, clinical- and device-related factors associated with SCS failure after permanent implantation. SCS failure was defined using a composite outcome comprising completed or planned explantation, inadequate pain relief accompanied by a desire or plan for removal, and documented device non-use. Secondary objectives were to estimate the prevalence of overall failure and explantation, describe reasons for explantation and duration of device use before removal, examine the associations of paresthesia or dysesthesia with generator type and implantable pulse generator location, and evaluate changes in pain intensity and morphine milligram equivalent dose after implantation.

## 2. Materials and Methods

### 2.1. Study Design and Setting

This retrospective, single-center cohort study was conducted at the Center Hospitalier de l’Université de Montréal (CHUM), a tertiary academic center in Montréal, Québec, Canada, providing specialized chronic pain and neurosurgical services. The study evaluated patients who underwent permanent spinal cord stimulation (SCS) implantation between 2007 and 2022. Reporting followed the Strengthening the Reporting of Observational Studies in Epidemiology (STROBE) recommendations [17].

### 2.2. Patient Identification and Eligibility

Potentially eligible patients were identified from the CHUM administrative database using Canadian Classification of Health Interventions (CCI) codes corresponding to permanent SCS implantation. Electronic medical records were subsequently reviewed to confirm eligibility and distinguish permanent implantation from trial stimulation, device revision, generator replacement, and explantation.

Patients were eligible if they were 18 years or older, underwent permanent SCS implantation at CHUM during the study period, and had at least six months of available clinical documentation before and after implantation. Patients whose permanent system had been implanted outside CHUM were excluded. Patients who underwent an unsuccessful or inconclusive trial and did not proceed to permanent implantation were also excluded. The study therefore evaluated outcomes after permanent implantation rather than failure across the complete SCS treatment pathway. The final cohort comprised 108 patients.

### 2.3. Preimplantation Assessment and Treatment

Before SCS was considered, patients received conservative treatments appropriate to their clinical condition, including pharmacological therapy, physical rehabilitation, and interventional pain procedures. Patients with refractory chronic pain underwent multidisciplinary assessment. Psychological evaluation was routinely performed, and psychiatric consultation was obtained when clinically indicated to identify active psychiatric or behavioral conditions that could adversely affect treatment adherence or outcomes.

The principal indications for implantation were persistent spinal pain syndrome after surgery, historically termed failed back surgery syndrome, and complex regional pain syndrome. Other indications included phantom limb pain, neuropathic pain, and refractory non-operated lumbosciatica.

All included patients underwent trial stimulation before permanent implantation. Permanent implantation was performed using either a percutaneous or surgical approach. The treating clinical team selected the implantation technique, lead configuration, generator type, stimulation settings, and implantable pulse generator (IPG) location according to clinical and technical considerations. Device manufacturer and model information were not consistently available in the retrospective records and therefore could not be reported reliably.

### 2.4. Data Collection

Clinical, procedural, and outcome information was retrospectively extracted from electronic medical records and entered into a standardized database. The following variables were collected:

- age and sex;
- marital status and educational level;
- self-reported comfort with smartphone use;
- tobacco, alcohol, cannabis, and cocaine use;
- pain diagnosis, anatomical location, and duration before implantation;
- medical and psychiatric history;
- previous lumbar surgery;
- implantation approach;
- number of leads and electrodes;
- generator type, categorized as rechargeable or non-rechargeable;
- IPG location;
- device revisions and complications;
- unwanted paresthesia or dysesthesia;
- device-use status;
- explantation status and reasons for explantation;
- duration of device use;
- pain intensity before and after implantation;
- opioid exposure before and after implantation.

Comorbidity burden was defined as the number of documented prespecified conditions, comprising diabetes mellitus, hypertension, hypercholesterolemia, obesity, chronic kidney disease, cancer, and previous lumbar surgery. Previous lumbar surgery was also examined separately as a clinical characteristic. This study-specific comorbidity count was not equivalent to a validated index such as the Charlson Comorbidity Index and was therefore interpreted as exploratory.

### 2.5. Outcome Definitions

#### 2.5.1. Primary Outcome

The primary outcome was SCS failure after permanent implantation. Failure was defined as the presence of one or more of the following:

- completed urgent or elective explantation;
- a documented decision or plan for explantation;
- clinician-documented inadequate pain relief accompanied by a desire or plan for device removal;
- documented discontinuation or non-use of the device at the most recent clinical assessment.

A uniform percentage reduction in pain was not used to define inadequate pain relief because such information was not consistently available in the retrospective medical records. Outcome classification was therefore based on the treating clinician’s documentation and the patient’s reported treatment benefit and device use.

Ongoing treatment success was defined as retention and continued use of the implanted system without meeting any component of the failure definition at the most recent documented follow-up. Because the composite outcome included clinically heterogeneous events, completed explantation, planned explantation, inadequate pain relief, and device non-use were also evaluated descriptively as separate components.

Because more than one component could be documented for an individual patient, failure components were classified hierarchically for descriptive reporting to prevent double-counting. Patients were assigned sequentially to: (1) completed explantation, (2) documented planned explantation, (3) documented device non-use, and (4) inadequate pain relief or loss of efficacy accompanied by a desire or plan for removal. Each patient was assigned to the first applicable category.

#### 2.5.2. Secondary Outcomes

Secondary outcomes included:

- prevalence and documented reasons for explantation;
- duration of device use before explantation;
- frequency of device revision, complications, and adverse effects;
- occurrence of unwanted paresthesia or dysesthesia;
- associations of paresthesia or dysesthesia with generator type and IPG location;
- change in pain intensity following implantation;
- change in opioid exposure expressed as daily morphine milligram equivalents (MME).

Reasons for explantation were not mutually exclusive; therefore, an individual patient could have more than one documented reason.

Complications, adverse effects, and treatment-related events were abstracted independently of the composite failure classification. These events were not mutually exclusive from one another or from the failure components; therefore, an individual patient could be represented in both classifications. “No pain relief” and “loss of efficacy” were recorded whenever documented as clinical events. They contributed to composite failure when associated with completed or planned explantation or when accompanied by a documented desire or plan for device removal. Documented device non-use directly met the composite failure definition. Revision was defined as a documented non-routine surgical or device-related reintervention; routine pulse-generator replacement for expected battery depletion was excluded.

### 2.6. Pain and Opioid Outcomes

Pain intensity was measured using a 0–10 visual analog scale (VAS), with higher scores indicating greater pain. Baseline VAS was defined as the most recent available score recorded before phase 1 trial stimulation. Opioid exposure was expressed as daily morphine milligram equivalents (MME), and baseline MME was defined as the most recent documented opioid regimen before permanent implantation. Prescribed opioid doses were converted using the 2015 Washington State Agency Medical Directors’ Group opioid conversion guidance [18]. Thus, VAS and MME had different baseline anchors because measurements obtained at a common preimplantation time point were not consistently available in the retrospective records. For both outcomes, the postimplantation value was the measurement documented at the study assessment or most recent available follow-up. Postimplantation assessment intervals were not standardized.

### 2.7. Follow-Up

Follow-up began on the date of permanent SCS implantation. Clinical records were reviewed until the first documented occurrence of SCS failure or the most recent available clinical contact. For patients who underwent explantation, device-use duration was calculated from permanent implantation to device removal. Patients without documented failure were classified by treatment status at the most recent clinical assessment.

Although implantation and explantation dates were available, exact dates of onset were not consistently documented for the non-explantation components of the composite outcome, including inadequate pain relief, planned removal, and device non-use. Complete censoring dates were also unavailable for some patients. Consequently, reliable time-to-event analysis could not be performed.

### 2.8. Statistical Analysis

Data were entered into Microsoft Excel for Microsoft 365 (Microsoft Corporation, Redmond, WA, USA), reviewed for completeness and internal consistency, and analyzed using IBM SPSS Statistics, version 26.0 (IBM Corp., Armonk, NY, USA). Categorical variables were summarized as frequencies and percentages. Continuous variables were summarized using means and standard deviations when approximately normally distributed and medians and interquartile ranges when distributions were skewed. Minimum and maximum values were reported where clinically informative.

Patients with and without SCS failure were compared using Pearson’s chi-squared test. Fisher’s exact test or the Monte Carlo exact test was used when expected cell counts were small. Risk ratios (RRs) with 95% confidence intervals (CIs) were calculated for the principal binary associations involving sex and smoking. Because comorbidity count was analyzed as a six-category variable and demonstrated a nonmonotonic relationship with failure, Cramér’s *V*, with its 95% CI, was reported as the omnibus effect size. To address multiple comparisons, the Benjamini–Hochberg procedure was applied across the 26 association tests included in the analysis, and false-discovery-rate-adjusted *q*-values were calculated.

Non-normally distributed continuous variables were compared between independent groups using the Mann–Whitney *U* test. Paired pre- and post-implantation VAS and MME measurements were compared using the Wilcoxon signed-rank test. Within-patient changes were summarized as medians and interquartile ranges. VAS change was calculated as baseline minus follow-up, such that positive values indicated pain reduction, whereas MME change was calculated as follow-up minus baseline.

Values recorded as “not documented,” “unknown,” or “not available” were treated as missing rather than as exposure categories. Primary association analyses were conducted using complete cases, and variable-specific denominators were reported. For variables with missing observations, composite-failure proportions were compared between patients with and without recorded data using Fisher’s exact test as a sensitivity analysis. The missingness mechanism could not be established from the retrospective records. Complete-case analyses therefore assumed that, within each univariable comparison, missingness was not materially dependent on the outcome or unobserved exposure values. Although the sensitivity analyses did not identify significant differences in failure according to data availability, these comparisons had limited power and could not exclude informative missingness. No statistical imputation was performed.

Failure components were summarized using the mutually exclusive hierarchical classification described above. Component-specific predictor analyses were not performed because several subgroups contained very few patients, resulting in sparse cells and unreliable effect estimates.

All statistical tests were two-sided, and an unadjusted *p*-value below 0.05 was considered statistically significant. Because the analyses were exploratory and involved multiple comparisons, findings were interpreted with attention to effect magnitude, precision, clinical relevance, and false-discovery-rate-adjusted results.

### 2.9. Ethical Approval

The study was conducted in accordance with the Declaration of Helsinki and was approved by the CHUM Research Ethics Board, Montréal, Québec, Canada (approval No. 2024-11698 [23.154], 7 December 2023). The requirement for individual informed consent was waived by the Research Ethics Board because the study involved retrospective analysis of existing clinical records.

### 2.10. Data Availability

The patient-level dataset contains protected clinical information and is therefore not publicly available. Deidentified data and associated analytical materials may be made available by the corresponding author upon reasonable request, subject to approval by the CHUM Research Ethics Board and applicable institutional privacy and data-sharing requirements.

## 3. Results

### 3.1. Patient and Treatment Characteristics

A total of 108 adults who underwent permanent spinal cord stimulation (SCS) implantation were included. The mean age was 62.9 ± 12.7 years (range, 27–90 years), and 60 patients (55.6%) were male. Most patients had experienced pain for more than 5 years before implantation (86/108, 79.6%), 74 (68.5%) had undergone previous surgery, and 40 (37.0%) were current smokers (Table 1).

**Figure 1:**
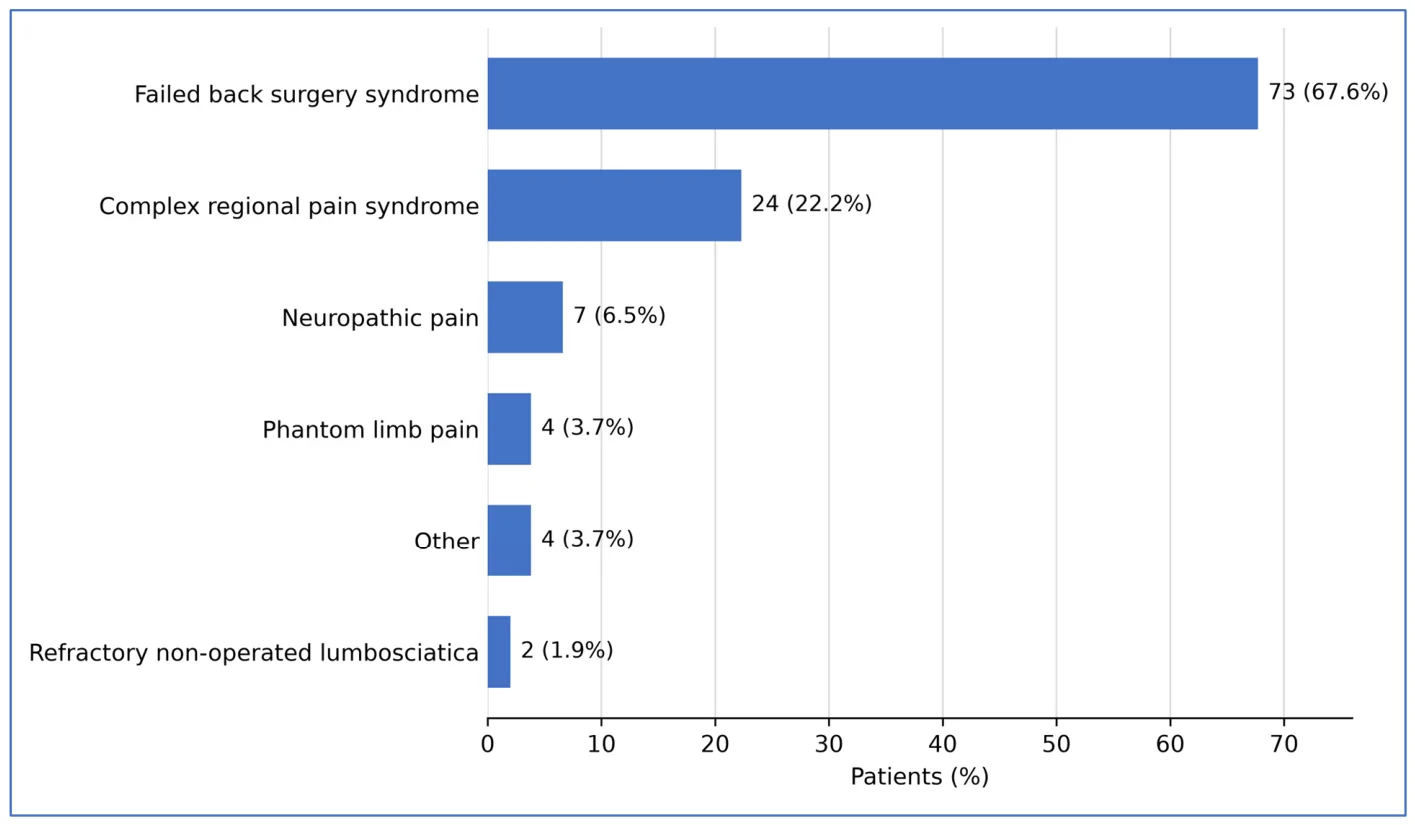
Pain diagnoses for which spinal cord stimulation was implanted (*n* = 108). Values are presented as *n* (%). Diagnoses were not mutually exclusive; therefore, percentages sum to more than 100%.

The most frequently recorded indication was failed back surgery syndrome, also termed persistent spinal pain syndrome after surgery (73 patients, 67.6%), followed by complex regional pain syndrome (24 patients, 22.2%). Diagnostic categories were not mutually exclusive (Figure 1). Among patients with a documented implantation approach, 93/105 (88.6%) underwent percutaneous implantation, and 12/105 (11.4%) underwent surgical implantation. Smartphone-comfort information was unavailable for 17 patients (15.7%). Implantation approach was unavailable for 3 (2.8%), generator type for 18 (16.7%), IPG location for 4 (3.7%), and the numbers of leads and electrodes for 4 (3.7%) each. The implantable pulse generator (IPG) was most frequently located in the abdomen.

### 3.2. SCS Failure and Explantation

**Figure 2:**
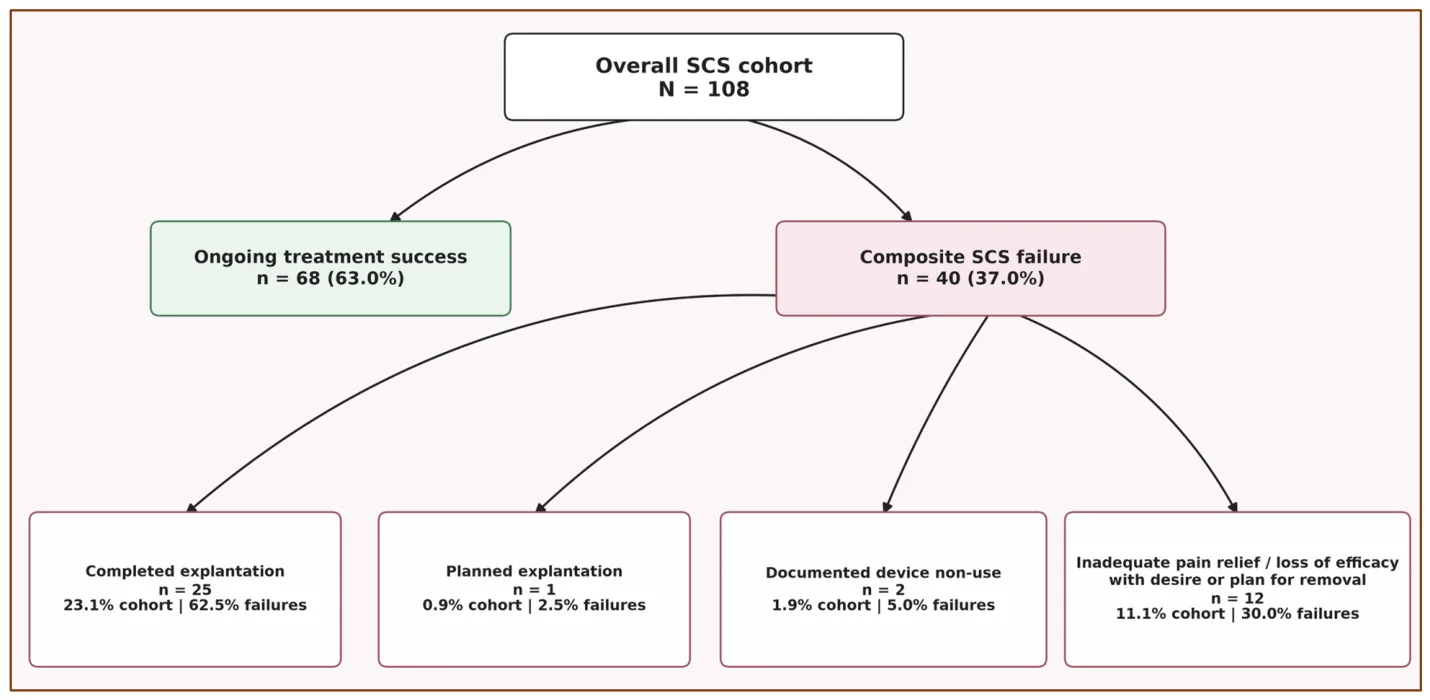
Composite Spinal Cord Stimulation Outcomes and Mutually Exclusive Components of Failure. SCS, spinal cord stimulation. Failure components were classified hierarchically and assigned to the first applicable category. Percentages for each component are reported relative to the overall cohort (*N* = 108) and all composite failures (*N* = 40), respectively. Component counts sum to 40; percentages may not total 100% because of rounding.

At the most recent assessment, 40 of 108 patients (37.0%) met the composite definition of SCS failure, whereas 68 patients (63.0%) had ongoing treatment success. Using the hierarchical, mutually exclusive classification, 25 patients underwent completed explantation (23.1% of the cohort; 62.5% of failures), one had a documented plan for explantation (0.9%; 2.5%), two had documented device non-use without completed or planned explantation (1.9%; 5.0%), and 12 had inadequate pain relief or loss of efficacy accompanied by a desire for device removal without meeting a higher-priority component (11.1%; 30.0%). The mutually exclusive components of the composite failure outcome are presented in Figure 2.

**Figure 3:**
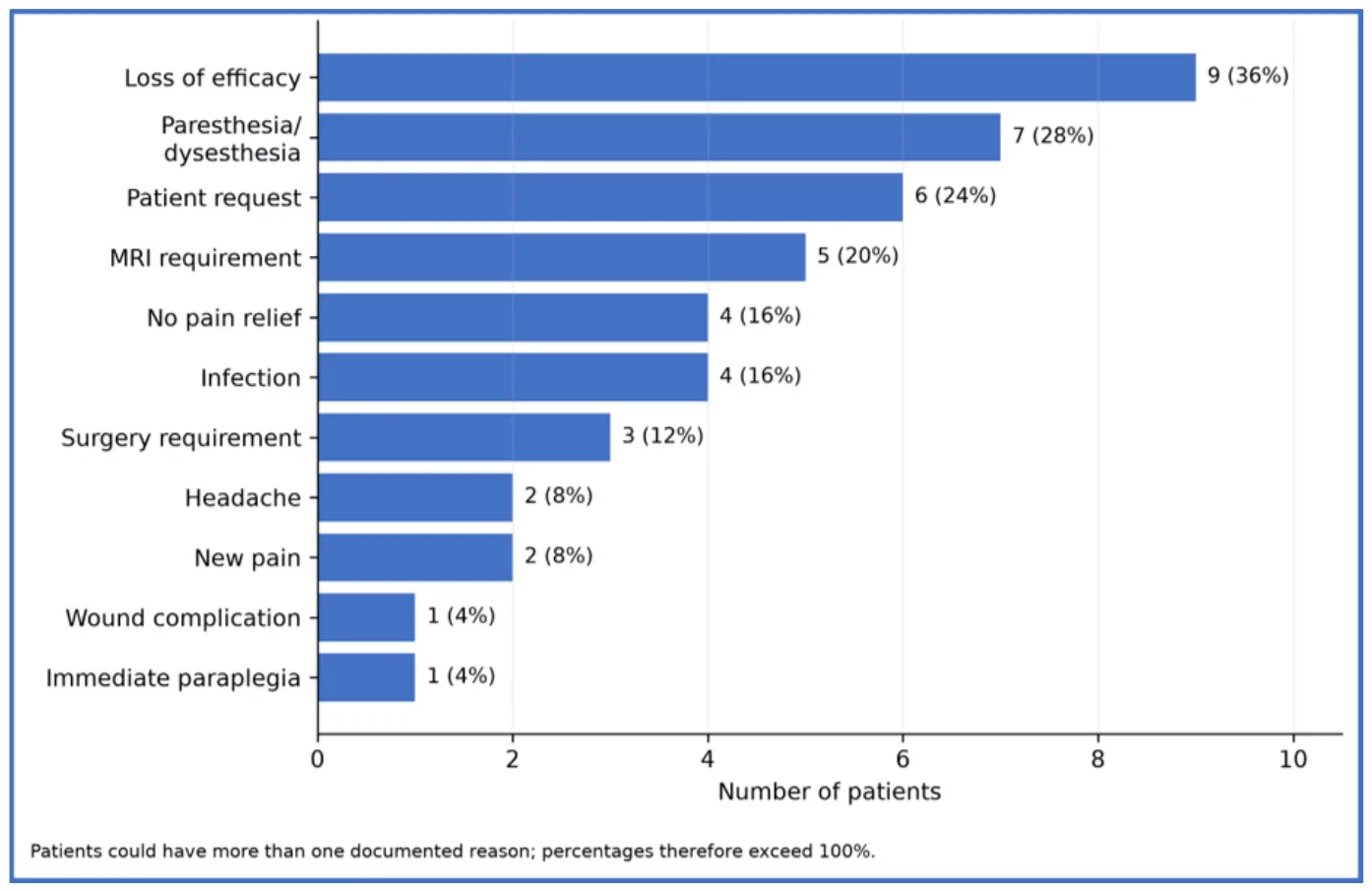
Documented reasons for spinal cord stimulation explantation among 25 patients. Values are presented as number and percentage of explanted patients. More than one reason could be recorded for each patient; therefore, categories were not mutually exclusive. MRI, magnetic resonance imaging.

The most frequently documented reasons for explantation were loss of efficacy (9/25, 36.0%), unwanted paresthesia or dysesthesia (7/25, 28.0%), patient request (6/25, 24.0%), and the need for magnetic resonance imaging (5/25, 20.0%). Reasons were not mutually exclusive (Table 2 and Figure 3). Because follow-up duration varied among patients, these findings represent the prevalence of documented failure and explantation at the most recent assessment rather than standardized incidence or cumulative-risk estimates.

Among the 25 explanted patients, the mean duration of device use was 124.7 ± 76.3 months; the median was 84 months (Q1–Q3, 66–200 months), and the range was 0.03–204 months.

### 3.3. Complications and Adverse Effects

**Figure 4:**
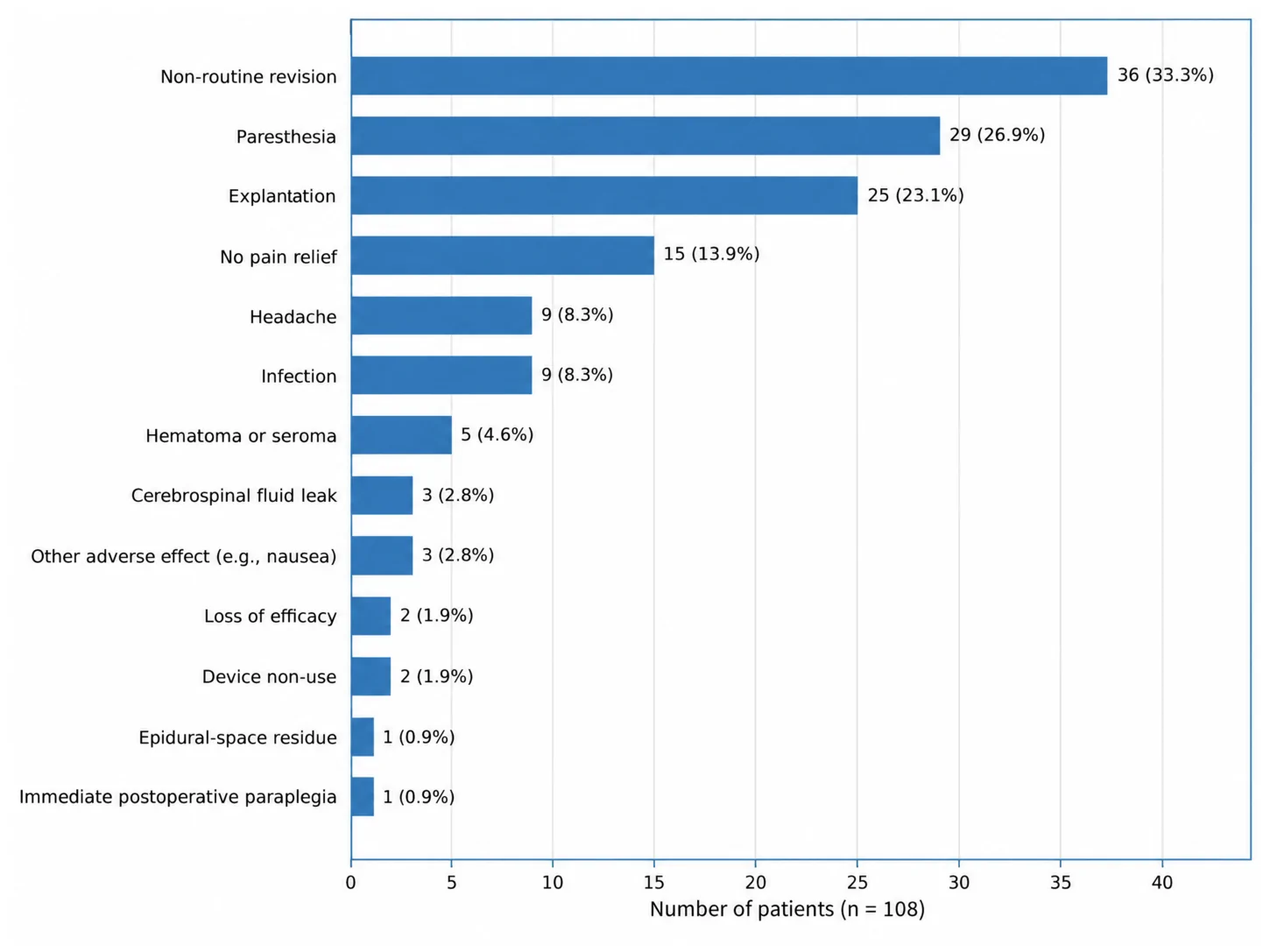
Documented treatment-related events, complications, and adverse effects following permanent spinal cord stimulation implantation (*n* = 108). Documented treatment-related events, complications, and adverse effects following permanent spinal cord stimulation implantation (*n* = 108). Values represent the number and percentage of the full cohort. Categories were not mutually exclusive, and an individual patient could contribute to more than one category. These events were abstracted independently of the composite failure classification and could therefore overlap with the failure components shown in Figure 2. “No pain relief” and “loss of efficacy” contributed to composite failure when associated with completed or planned explantation or accompanied by a documented desire or plan for device removal, whereas documented device non-use directly met the failure definition. “Non-routine revision” was defined as a documented non-routine surgical or device-related reintervention; routine pulse-generator replacement for expected battery depletion was excluded. One patient developed immediate postoperative paraplegia associated with an epidural hematoma and was therefore represented in both the “Immediate postoperative paraplegia” and “Hematoma or seroma” categories. “Other adverse effects” included nausea. CSF, cerebrospinal fluid; SCS, spinal cord stimulation.

Device revision was the most frequently documented treatment-related event, occurring in 36 patients (33.3%). Routine pulse-generator replacement for expected battery depletion was excluded from the revision category. The categories were recorded independently, were not mutually exclusive, and could overlap with the components of the composite failure outcome. Unwanted paresthesia occurred in 29 (26.9%), no pain relief in 15 (13.9%), infection in 9 (8.3%), and headache in 9 (8.3%). Patients could have more than one complication or adverse effect (Figure 4).

One patient developed immediate postoperative paraplegia associated with an epidural hematoma identified on postoperative day 1. The device was urgently explanted within one day of implantation. Information regarding the subsequent degree of neurological recovery was not sufficiently documented in the available records.

### 3.4. Factors Associated with SCS Failure

Table 3 presents the univariable comparisons. Failure occurred in 21/48 women (43.8%) and 19/60 men (31.7%). Female sex was associated with an estimated 38% higher risk of failure than male sex, although the confidence interval was wide and the association was not statistically significant (RR = 1.38, 95% CI 0.85–2.26; *p* = 0.196). Failure occurred in 20/40 smokers (50.0%) and 20/68 nonsmokers (29.4%). Smoking was associated with a higher unadjusted risk of failure (RR = 1.70, 95% CI 1.05–2.75; *p* = 0.032). This association relates to the composite failure outcome and should not be attributed to any individual failure component. Component-specific analyses were not performed because of the small subgroup sizes.

The distribution of failure differed across the six comorbidity-count categories (Cramér’s *V* = 0.34, 95% CI 0.00–0.48; omnibus Monte Carlo *p* = 0.019). However, failure proportions did not increase monotonically with increasing comorbidity count. The finding therefore indicates overall heterogeneity among categories rather than a linear dose–response relationship.

Smartphone-comfort information was documented for 91 patients. After excluding the 17 patients with undocumented information, failure occurred in 7/23 patients (30.4%) who were not at all comfortable with smartphones, 17/40 (42.5%) with average comfort, and 9/28 (32.1%) with high comfort. The association was not statistically significant (*p* = 0.544). In a sensitivity analysis, failure did not differ significantly between patients with documented and undocumented smartphone-comfort information (36.3% versus 41.2%; Fisher’s exact *p* = 0.786).

Failure status did not differ significantly between patients with and without recorded generator type (*p* = 0.594), implantation approach (*p* = 0.294), IPG location (*p* = 0.626), or smartphone-comfort information (*p* = 0.786). These sensitivity analyses were limited by the small missing-data groups. No statistically significant associations were observed for marital status, educational level, previous surgery, pain location, pain duration, psychiatric history, diagnosis, implantation approach, generator type or location, number of leads or electrodes, alcohol use, cannabis use, or cocaine use (all *p* > 0.05). These analyses were unadjusted.

A total of 26 association tests were included in the Benjamini–Hochberg correction. Neither smoking nor comorbidity count remained statistically significant after correction (both *q* = 0.416). Therefore, these associations should be interpreted as exploratory.

### 3.5. Paresthesia and Device Characteristics

Among 90 patients with a documented generator type, paresthesia occurred in 15/46 (32.6%) with rechargeable generators and 11/44 (25.0%) with non-rechargeable generators (*p* = 0.426; Table 4). Paresthesia occurred in 19/69 patients (27.5%) with an abdominal IPG and 10/37 (27.0%) with a back or flank IPG (*p* = 0.955; Table 5).

### 3.6. Pain Intensity and Opioid Exposure

Paired VAS measurements were available for 102 patients. Mean pain intensity decreased from 8.4 ± 1.3 before trial stimulation to 5.5 ± 2.6 at follow-up; the corresponding medians were 8.0 (Q1–Q3, 7.0–10.0) and 6.0 (Q1–Q3, 3.25–7.75), respectively. The median within-patient VAS reduction, calculated as baseline minus follow-up, was 2.50 points (IQR, 1.00–5.00). The reduction was statistically significant (Wilcoxon signed-rank test: 80 decreases, 5 increases, and 17 ties; *Z* = −7.68; exact two-sided *p* < 0.001; Table 6).

Complete paired MME measurements were available for 108 patients. Median daily opioid exposure decreased from 67.5 MME (Q1–Q3, 0–180) before implantation to 60.0 MME (Q1–Q3, 0–180) after implantation. The median within-patient change, calculated as follow-up minus baseline, was 0 MME/day (IQR, −60 to 40). Among the 108 pairs, 44 showed a decrease, 37 an increase, and 27 no change. The paired difference was not statistically significant (Wilcoxon signed-rank test: *Z* = −0.46; exact two-sided *p* = 0.649; Table 7).

The VAS and MME analyses had different baseline anchors, and postimplantation measurements were not obtained at a standardized interval after implantation. Outcome-specific assessment dates and measurements at a uniform follow-up time point were not consistently available; therefore, the distribution of implantation-to-assessment intervals and a fixed-time-point sensitivity analysis could not be reported reliably.

## 4. Discussion

### 4.1. Principal Findings

In this retrospective, single-center cohort of 108 patients who underwent permanent spinal cord stimulation (SCS) implantation, 40 patients (37.0%) met the composite definition of treatment failure, whereas 68 (63.0%) had ongoing treatment success at their most recent assessment. Completed explantation occurred in 25 patients (23.1%). Among the remaining 15 failures, one involved planned explantation, two involved documented device non-use, and 12 involved inadequate pain relief or loss of efficacy accompanied by a desire or plan for removal. Loss of efficacy was the most frequently documented reason for explantation, followed by unwanted paresthesia or dysesthesia and patient request. Revision was the most common treatment-related event, and approximately one-quarter of the cohort experienced unwanted paresthesia. These findings are consistent with previous reports identifying loss of therapeutic benefit as a major reason for SCS explantation [7,8,9,10].

Smoking was associated with failure in the unadjusted analysis, but the association did not remain statistically significant after correction for multiple comparisons and should therefore be considered exploratory. The observed association between smoking and SCS failure applies to the composite outcome. Because the individual failure components contained few events, component-specific associations could not be estimated reliably. The present analysis therefore cannot determine whether smoking was associated specifically with loss of efficacy, device non-use, planned removal, or completed explantation. However, this association should not be interpreted as causal because smoking may represent or coexist with other unmeasured socioeconomic, behavioral, and clinical factors. The distribution of failure also differed across comorbidity-count categories, although the pattern was not monotonic and did not demonstrate a dose–response relationship.

Female sex was not significantly associated with failure in this cohort. Previous studies have reported inconsistent findings regarding the association between biological sex and SCS outcomes [12,19]. No significant associations were observed for psychiatric history, pain duration, educational level, comfort with smartphone use, pain diagnosis, implantation approach, or the evaluated device characteristics. These findings are broadly consistent with the heterogeneity of clinical predictors reported in the literature [3,4,11,12,13,14].

Mean pain intensity decreased from 8.4 before trial stimulation to 5.5 at the postimplantation assessment. Although this represents a substantial cohort-level improvement, the uncontrolled pre–post design prevents attribution of the observed change exclusively to SCS. Daily morphine milligram equivalent (MME) doses did not change significantly, and their distribution showed considerable interindividual variability.

### 4.2. Interpretation of SCS Failure and Explantation

The composite failure prevalence of 37.0% exceeded the explantation prevalence of 23.1%, demonstrating that explantation alone did not capture all clinically unsuccessful treatments. Previous studies have frequently used explantation as an objective endpoint because it can be identified relatively reliably from clinical and administrative records [7,8,9,10,20,21]. However, patients may retain an implanted system despite inadequate analgesia or may discontinue device use without undergoing removal. A broader outcome definition may therefore capture clinically unsuccessful therapy more comprehensively.

Nevertheless, the composite outcome encompassed clinically heterogeneous events. Using a hierarchical classification, we separately reported completed explantation, planned explantation, device non-use, and inadequate pain relief or loss of efficacy accompanied by a desire or plan for device removal (Figure 2). These components may have different determinants and should not be interpreted as reflecting a single biological or technical mechanism of failure. Component-specific predictor analyses were not considered reliable because planned explantation and device non-use occurred in only one and two patients, respectively. Accordingly, the observed association with smoking applies to the composite outcome and cannot be attributed to a specific failure mechanism. The retrospective records did not consistently document the duration of initial benefit before loss of efficacy, whether paresthesia or dysesthesia developed immediately or later, or the detailed motivations underlying patient-requested removal and device non-use. Consequently, these temporal patterns and motivations could not be characterized reliably.

Loss of efficacy was the most frequently documented reason for explantation, consistent with previous studies of SCS withdrawal and device removal [8,9,10,20,21]. The need for magnetic resonance imaging also contributed to explantation in this cohort. This finding should be interpreted in the context of the 2007–2022 study period, during which MRI compatibility and device technology evolved considerably [22,23].

The mean duration of device use before explantation was approximately 125 months, but the distribution was highly dispersed. Because patients were implanted over a 15-year period, they had substantially different opportunities to experience failure. The reported proportion therefore represents the prevalence of documented failure at the most recent assessment rather than a standardized incidence rate. Previous survival analyses have demonstrated the importance of accounting for observation time when estimating long-term explantation risk [8,9].

### 4.3. Patient-Related Factors Associated with Failure

Smoking was associated with failure in the unadjusted analysis, but the association did not remain statistically significant after correction for multiple comparisons and should therefore be considered exploratory. This finding is consistent with previous reports of reduced SCS effectiveness among smokers [11,24]. Potential explanations include impaired wound healing, vascular effects, greater pain burden, and differences in treatment adherence. However, smoking status may also be associated with socioeconomic circumstances, psychiatric characteristics, and other clinical factors that were not fully measured or adjusted for in this study. The result supports smoking cessation as a reasonable component of preimplantation optimization but does not establish smoking as an independent causal determinant or absolute contraindication to SCS.

The distribution of failure differed significantly across comorbidity-count categories, but the pattern was not monotonic. For example, patients with three comorbidities did not have a higher failure proportion than patients with one or two. The omnibus result therefore indicates heterogeneity among categories rather than a progressive increase in failure risk. Moreover, the study-specific count combined conditions with potentially different clinical consequences and included previous lumbar surgery, which was also examined separately. Because this measure was not equivalent to a validated comorbidity index, the finding should be considered exploratory and should not be used as a rigid patient-exclusion criterion.

Failure was numerically more frequent among women than men, but the difference was not statistically significant. Previous research concerning sex-related differences in SCS outcomes has produced mixed findings [12,19]. Such inconsistencies may reflect variation in pain diagnoses, psychosocial characteristics, treatment paradigms, follow-up duration, and outcome definitions. The present study may also have lacked sufficient statistical power to identify a modest association.

No significant association was found between documented psychiatric history and SCS failure. This finding should not be interpreted as evidence that psychological factors are unimportant. Patients underwent psychological evaluations before permanent implantation, and patients with active contraindications were unlikely to proceed to implantation. This selection process may have restricted the range and severity of psychiatric conditions represented in the cohort. Current recommendations continue to support comprehensive psychological and psychosocial assessment rather than reliance on any single psychiatric diagnosis [3,4].

Educational level and comfort with smartphone use were also not associated with failure. Smartphone comfort is not a validated measure of the ability to manage an implanted SCS system. Future research should evaluate more direct measures, including health literacy, device competency, charging and programming adherence, social support, and access to follow-up services.

### 4.4. Device Characteristics, Paresthesia, and Complications

Revision was the most frequently documented treatment-related event, occurring in approximately one-third of the cohort. Revisions may be required because of lead migration, hardware malfunction, battery-related procedures, wound complications, or attempts to restore therapeutic coverage. Technical complications and reoperations represent important components of the long-term treatment burden associated with SCS [10,15,21,25].

The safety findings also warrant consideration. Infection was documented in 9 patients (8.3%), which is higher than the approximately 3–5% commonly reported in the SCS literature. However, direct comparisons should be interpreted cautiously because infection definitions, surveillance periods, patient characteristics, and implantation practices vary among studies. Four infections resulted in explantation, indicating that not all documented infections required device removal. In addition, one patient developed immediate postoperative paraplegia associated with an epidural hematoma identified on postoperative day 1. The device was urgently explanted within one day of implantation; however, the subsequent degree of neurological recovery was not sufficiently documented. This rare but catastrophic complication is consistent with acute compression of the neural elements by an epidural hematoma and underscores the importance of immediate neurological assessment, urgent imaging, and prompt surgical management when a new neurological deficit occurs following SCS implantation.

Unwanted paresthesia occurred in 26.9% of patients and was not significantly associated with generator type or implantable pulse generator location. Nevertheless, an absence of statistical significance does not establish equivalence, particularly given the relatively small groups and incomplete generator data.

Generator type and location are also indirect indicators of the technical factors that may influence paresthesia. Stimulation waveform, amplitude, pulse width, frequency, lead position, programming strategy, and changes in body position may be more directly relevant but were not consistently available in the records [25,26]. Newer paradigms, including high-frequency and other paresthesia-free stimulation approaches, may provide alternatives for selected patients experiencing inadequate analgesia or intolerable paresthesia with conventional stimulation [16].

### 4.5. Pain Intensity and Opioid Exposure

Pain intensity decreased significantly after implantation. This finding is consistent with evidence supporting SCS for selected chronic neuropathic pain conditions [1,2,6]. However, the analysis was an uncontrolled pre–post comparison and could not distinguish the effect of SCS from concurrent treatment changes, natural symptom variation, regression to the mean, or differences in assessment timing. Furthermore, improvement at the cohort level can coexist with inadequate benefit or treatment failure among individual patients.

Daily MME did not change significantly following implantation. Although the median decreased from 67.5 to 60 MME, the mean increased and variability was substantial, indicating a highly skewed distribution. Pain improvement therefore was not accompanied by a consistent reduction in opioid exposure across the cohort. Previous studies have reported heterogeneous opioid trajectories following SCS, and higher preimplantation opioid exposure has been associated with poorer long-term outcomes in some populations [14,27].

Interpretation of the opioid findings is limited by the retrospective use of prescription records, potential changes in prescribing practices over the study period, and the absence of reliable information regarding adherence. Non-opioid analgesic use could not be quantified consistently. Although doses were converted using the prespecified AMDG guidance [18], differences in conversion methods alone should not be assumed to explain discrepancies between this study and previous research.

The VAS and MME comparisons also had different baseline anchors, and follow-up measurements were obtained at variable, nonstandardized intervals. Consequently, the observed changes may have been influenced by assessment timing, regression to the mean, concurrent treatment changes, and temporal changes in clinical practice. Measurements at a uniform time point, such as 12 months after implantation, were not consistently available; therefore, a fixed-time-point sensitivity analysis could not be performed.

### 4.6. Clinical Implications

The findings emphasize the importance of continued follow-up after permanent SCS implantation. A successful trial and subsequent implantation do not guarantee durable benefit, and treatment failure may become evident several years later. Structured follow-up should assess pain relief, functional improvement, device use, charging and programming requirements, adverse effects, opioid exposure, and the patient’s evolving treatment goals [3,4,28].

Smoking cessation and optimization of modifiable health conditions are reasonable components of multidisciplinary care. However, the present results do not support rigid exclusion criteria based solely on smoking, sex, psychiatric history, or the study-specific comorbidity count. Implantation decisions should remain individualized and should integrate clinical indication, psychological readiness, expectations, functional goals, adherence capacity, social context, and technical feasibility [3,4].

### 4.7. Strengths and Limitations

A principal strength of this study was its broad definition of clinically unsuccessful SCS therapy. By including planned explantation, inadequate pain relief accompanied by a desire or plan for removal, and documented device non-use, the study captured outcomes that an explantation-only definition would have missed. It also examined multiple patients, psychiatric, procedural, and device-related domains within a standardized tertiary-center setting.

Several limitations must be considered. First, the retrospective design was vulnerable to incomplete documentation, missing data, and outcome misclassification. Inadequate pain relief and device non-use were determined from clinical documentation rather than standardized validated thresholds, and outcome classification was not independently adjudicated.

Second, missing data constituted an additional limitation. The missingness mechanism could not be established retrospectively, and complete-case analyses may be biased if data availability was related to unobserved patient characteristics or outcomes. Although sensitivity comparisons did not identify significant differences in composite failure between patients with and without recorded data, the small missing-data groups limited statistical power and did not establish that the data were missing completely at random. No imputation was performed.

Third, the analyses were primarily univariable. No adjusted effect estimates were available to address confounding among smoking, sex, age, comorbidity burden, diagnosis, treatment year, and device characteristics. Multiple comparisons also increased the possibility of chance findings. The associations involving smoking and comorbidity categories should therefore be considered exploratory.

The individual components of the composite failure outcome had insufficient event counts for reliable component-specific predictor analyses. In particular, planned explantation and documented device non-use were rare, and completed explantations occurred for heterogeneous reasons. Accordingly, associations identified for the composite outcome should not be attributed to any individual failure component.

Fourth, unequal follow-up duration was not incorporated into the statistical analysis. Patients implanted earlier had a longer opportunity to experience failure than those implanted near the end of the study period. Kaplan–Meier estimation and Cox proportional-hazards regression would have provided a more appropriate assessment if complete event and censoring dates had been available.

Fifth, the study included only patients who proceeded to permanent implantation after trial stimulation. It therefore evaluated failure after permanent implantation rather than failure across the complete SCS treatment pathway. This selection process may also have reduced the observed influence of psychiatric factors.

Sixth, devices, stimulation paradigms, MRI compatibility, surgical techniques, programming practices, and selection criteria changed substantially between 2007 and 2022. These temporal changes were not adjusted for. Information on manufacturer, stimulation waveform, precise lead location, lead migration, and programming parameters was also unavailable or inconsistently documented.

Finally, this was a relatively small study conducted at a single specialized center. Local patient-selection procedures, implantation practices, and follow-up pathways may differ from those used elsewhere, limiting generalizability.

### 4.8. Future Research

Future multicenter prospective studies should use standardized definitions of SCS success and failure and report individual failure components separately. Complete longitudinal dates would permit estimation of failure-free survival and adjusted time-to-event analyses. Models should account for implantation year, follow-up duration, device generation, stimulation waveform, diagnosis, smoking, opioid exposure, psychosocial characteristics, and validated measures of comorbidity.

Future studies should also evaluate outcomes beyond pain intensity, including physical functioning, quality of life, patient satisfaction, device adherence, social participation, and total analgesic exposure. Such research may help distinguish modifiable factors from markers of underlying disease complexity and improve individualized patient selection and long-term management.

## 5. Conclusions

At the most recent documented assessment, the prevalence of composite SCS failure was 37.0%, whereas the prevalence of completed explantation was 23.1%. Because follow-up duration differed substantially among patients, these proportions should not be interpreted as standardized incidence or cumulative-risk estimates. This difference indicates that explantation alone may underestimate clinically unsuccessful therapy because inadequate pain relief, planned removal, and device non-use also represent important outcomes. Loss of efficacy was the most frequently documented reason for explantation, and device revision was the most common treatment-related event.

Smoking was associated with failure in the unadjusted analysis, but the association did not remain statistically significant after correction for multiple comparisons and should therefore be considered exploratory. Confirmation using adequately powered, prespecified multivariable and time-to-event analyses is required. Although failure also differed across comorbidity-count categories, the absence of a monotonic pattern precluded interpretation as a dose–response relationship. Female sex, psychiatric history, pain duration, implantation approach, and the evaluated device characteristics were not significantly associated with failure. Pain intensity improved significantly after implantation, whereas opioid exposure did not change significantly.

These findings support comprehensive patient assessment, optimization of modifiable health factors, and structured long-term follow-up after implantation. However, the retrospective design, heterogeneous composite outcome, unadjusted analyses, unequal follow-up duration, and single-center sample preclude causal or independent risk-factor conclusions. Prospective multicenter studies using standardized outcome definitions and adjusted time-to-event analyses are needed to identify determinants of durable SCS benefit and distinguish the mechanisms underlying treatment failure.

## Figures and Tables

**Table 1:** Baseline demographic and clinical characteristics of the study population (*n* = 108).

Characteristic	Category	Value, *n* (%)
Sex	Female	48 (44.4)
	Male	60 (55.6)
Age, years	Mean ± SD	62.9 ± 12.7
	Median (IQR)	62.1 (20.1)
	Range	27–90
Marital status	Single	20 (18.5)
	Married	37 (34.3)
	Divorced/separated	11 (10.2)
	Widowed	6 (5.6)
	Other	34 (31.4)
Educational level	Primary	4 (3.7)
	Secondary	43 (39.8)
	Technical school	4 (3.7)
	Post-secondary, non-tertiary	19 (17.6)
	University	22 (20.4)
	Other	16 (14.8)
Comfort with smartphone use	Not at all	23 (21.3)
	Average	40 (37.0)
	High	28 (25.9)
	Not documented	17 (15.7)
Number of comorbidities	0	45 (41.7)
	1	24 (22.2)
	2	18 (16.7)
	3	15 (13.9)
	4	5 (4.6)
	5	1 (0.9)
Previous surgery	Yes	74 (68.5)
	No	34 (31.5)
Substance use	Alcohol	15 (13.9)
	Tobacco	40 (37.0)
	Cannabis	8 (7.4)
	Cocaine	1 (0.9)
Pain location	Lower extremity	52 (48.1)
	Lower extremity and low back	39 (36.1)
	Upper extremity	13 (12.0)
	Other	4 (3.7)
Pain duration before implantation	<2 years	2 (1.9)
	2–5 years	20 (18.5)
	>5 years	86 (79.6)

Data are presented as *n* (%) unless otherwise indicated. The study-specific comorbidity counts included diabetes mellitus, hypertension, hypercholesterolemia, obesity, chronic kidney disease, cancer, and previous lumbar surgery. Previous lumbar surgery was also examined separately. IQR, interquartile range; SD, standard deviation.

**Table 2:** Prevalence, documented reasons, and duration of spinal cord stimulation explantation.

Measure	Value
Loss of efficacy	9 (36.0)
Paresthesia/dysesthesia	7 (28.0)
Patient request	6 (24.0)
Need for MRI	5 (20.0)
No pain relief	4 (16.0)
Infection	4 (16.0)
Need for surgery	3 (12.0)
Headache	2 (8.0)
New pain	2 (8.0)
Wound dehiscence/seroma/hematoma	1 (4.0)
Immediate paraplegia	1 (4.0)
Device-use duration, mean ± SD, months	124.7 ± 76.3
Device-use duration, median (Q1–Q3), months	84 (66–200)
Device-use duration, range, months	0.03–204

Explantation occurred in 25 of 108 patients (23.1%). Reasons are presented as *n* (%) of the 25 explanted patients. More than one reason could be documented; therefore, percentages exceed 100%. Device-use duration was calculated from permanent SCS implantation to explantation. MRI, magnetic resonance imaging; Q1, first quartile; Q3, third quartile; SD, standard deviation.

**Table 3:** Univariable associations between patient, clinical, and device characteristics and composite spinal cord stimulation failure.

Variable	Category	Failure, *n* (%)	Success, *n* (%)	*p*-Value	Effect Size (95% CI)
Sex	Female	21 (43.8)	27 (56.3)	0.196	RR = 1.38 (0.85–2.26)
	Male	19 (31.7)	41 (68.3)		
Marital status	Single	6 (30.0)	14 (70.0)	0.442 *	
	Married	17 (45.9)	20 (54.1)		
	Divorced/separated	6 (54.5)	5 (45.5)		
	Widowed	2 (33.3)	4 (66.7)		
	Other	9 (26.5)	25 (73.5)		
Educational level	Primary	2 (50.0)	2 (50.0)	0.413 *	
	Secondary	17 (39.5)	26 (60.5)		
	Technical school	1 (25.0)	3 (75.0)		
	Post-secondary, non-tertiary	9 (47.4)	10 (52.6)		
	University	4 (18.2)	18 (81.8)		
	Other	7 (43.8)	9 (56.3)		
Smartphone comfort (*n* = 91)	Not at all	7 (30.4)	16 (69.6)	0.544	
	Average	17 (42.5)	23 (57.5)		
	High	9 (32.1)	19 (67.9)		
Previous surgery	Yes	29 (39.2)	45 (60.8)	0.494	
	No	11 (32.4)	23 (67.6)		
Comorbidity count	0	11 (24.4)	34 (75.6)	0.019 *	Cramér’s V = 0.34 (0.00–0.48)
	1	14 (58.3)	10 (41.7)		
	2	9 (50.0)	9 (50.0)		
	3	3 (20.0)	12 (80.0)		
	4	2 (40.0)	3 (60.0)		
	5	1 (100.0)	0 (0.0)		
Pain location	Lower extremity	19 (36.5)	33 (63.5)	0.986 *	
	Lower extremity and low back	14 (35.9)	25 (64.1)		
	Upper extremity	5 (38.5)	8 (61.5)		
	Other	2 (50.0)	2 (50.0)		
Pain duration	<2 years	1 (50.0)	1 (50.0)	0.516 *	
	2–5 years	5 (25.0)	15 (75.0)		
	>5 years	34 (39.5)	52 (60.5)		
Anxiety	Yes	6 (33.3)	12 (66.7)	0.722	
	No	34 (37.8)	56 (62.2)		
Major depression	Yes	13 (36.1)	23 (63.9)	0.888	
	No	27 (37.5)	45 (62.5)		
Post-traumatic stress disorder	Yes	2 (20.0)	8 (80.0)	0.317 †	
	No	38 (38.8)	60 (61.2)		
CRPS	Yes	9 (37.5)	15 (62.5)	0.958	
	No	31 (36.9)	53 (63.1)		
Failed back surgery syndrome	Yes	28 (38.4)	45 (61.6)	0.682	
	No	12 (34.3)	23 (65.7)		
Phantom limb pain	Yes	0 (0.0)	4 (100.0)	0.294 †	
	No	40 (38.5)	64 (61.5)		
Neuropathic pain	Yes	2 (28.6)	5 (71.4)	1.000 †	
	No	38 (37.6)	63 (62.4)		
Refractory lumbosciatica	Yes	1 (50.0)	1 (50.0)	1.000 †	
	No	39 (36.8)	67 (63.2)		
Other diagnosis	Yes	2 (50.0)	2 (50.0)	0.626 †	
	No	38 (36.5)	66 (63.5)		
Approach (*n* = 105)	Percutaneous	37 (39.8)	56 (60.2)	0.366 †	
	Surgical	3 (25.0)	9 (75.0)		
Generator type (*n* = 90)	Rechargeable	18 (39.1)	28 (60.9)	0.469	
	Non-rechargeable	14 (31.8)	30 (68.2)		
Generator location (*n* = 104)	Abdominal	25 (36.2)	44 (63.8)	0.927 †	
	Back/flank	13 (37.1)	22 (62.9)		
Number of leads (*n* = 104)	1	20 (35.7)	36 (64.3)	0.534	
	2	20 (41.7)	28 (58.3)		
Number of electrodes (*n* = 104)	4	17 (43.6)	22 (56.4)	0.792 *	
	8	21 (35.0)	39 (65.0)		
	16	2 (40.0)	3 (60.0)		
Alcohol use	Yes	6 (40.0)	9 (60.0)	0.798	
	No	34 (36.6)	59 (63.4)		
Tobacco use	Yes	20 (50.0)	20 (50.0)	0.032	RR = 1.70 (1.05–2.75)
	No	20 (29.4)	48 (70.6)		
Cannabis use	Yes	3 (37.5)	5 (62.5)	1.000 †	
	No	37 (37.0)	63 (63.0)		
Cocaine use	Yes	0 (0.0)	1 (100.0)	1.000 †	
	No	40 (37.4)	67 (62.6)		

Percentages are row percentages. Failure was defined as completed or planned explantation, clinician-documented inadequate pain relief accompanied by a desire or plan for removal, or documented device non-use. Pearson’s chi-squared test was used unless otherwise indicated. * Monte Carlo exact test; † Fisher’s exact test. Analyses were based on complete cases, and variable-specific denominators are shown where applicable. Values recorded as “not documented,” “unknown,” or “not available” were treated as missing and excluded from the primary association analyses. Missing observations were as follows: smartphone comfort, 17; implantation approach, 3; generator type, 18; IPG location, 4; number of leads, 4; and number of electrodes, 4. RR, risk ratio; CI, confidence interval. Male sex was the reference category for the sex comparison, and no tobacco use was the reference category for the tobacco-use comparison. Cramér’s V represents the omnibus effect size for the six-category comorbidity-count comparison. Benjamini–Hochberg correction was applied across the 26 association tests reported in Table 3. Smoking and comorbidity count each had an adjusted q-value of 0.416 and therefore did not remain statistically significant after correction. Results are unadjusted and do not establish independent or causal associations. CRPS, complex regional pain syndrome.

**Table 4:** Association between generator type and unwanted paresthesia (*n* = 90).

Generator Type	Paresthesia, *n* (%)	No Paresthesia, *n* (%)	Total	*p*-Value
Rechargeable	15 (32.6)	31 (67.4)	46	0.426
Non-rechargeable	11 (25.0)	33 (75.0)	44	
Total	26 (28.9)	64 (71.1)	90	

Note: The *p*-value was calculated using Pearson’s chi-square test without continuity correction. Percentages are row percentages. The analysis included patients with documented generator type and paresthesia status. Pearson’s chi-squared test was used.

**Table 5:** Association between implantable pulse generator location and unwanted paresthesia (*n* = 106).

IPG Location	Paresthesia, *n* (%)	No Paresthesia, *n* (%)	Total	*p*-Value
Abdominal	19 (27.5)	50 (72.5)	69	0.955
Back/flank	10 (27.0)	27 (73.0)	37	

Percentages are row percentages. The analysis included patients with documented IPG location and paresthesia status. Pearson’s chi-squared test was used. IPG, implantable pulse generator.

**Table 6:** Paired VAS measurements.

Assessment	Paired *n*	Mean ± SD	Median (Q1–Q3)
Before implantation	102	8.44 ± 1.32	8.00 (7.00–10.00)
After implantation	102	5.49 ± 2.63	6.00 (3.25–7.75)
Paired VAS reduction (before − after)	102	-	2.50 (1.00–5.00)

Wilcoxon signed-rank test: 80 decreases, 5 increases, and 17 ties; 85 nonzero differences; Z = −7.68; exact two-sided *p* < 0.001.

**Table 7:** Paired MME measurements.

Assessment	Paired *n*	Mean ± SD	Median (Q1–Q3)
Before implantation	108	132.93 ± 211.22	67.50 (0–180.00)
After implantation	108	160.14 ± 375.21	60.00 (0–180.00)
Paired MME change (after − before)	108	-	0 (−60 to 40)

Wilcoxon signed-rank test: 44 decreases, 37 increases, and 27 ties; 81 nonzero differences; Z = −0.46; exact two-sided *p* = 0.649.

## Data Availability

The patient-level data contain protected health information and are not publicly available because of ethical and privacy restrictions. Deidentified data and associated analytical materials may be made available by the corresponding author upon reasonable request, subject to approval by the CHUM Research Ethics Board and applicable institutional data-sharing requirements.

## Abbreviations

The following abbreviations are used in this manuscript:

Abbreviation	Definition
AMDG	Agency Medical Directors’ Group
CCI	Canadian Classification of Health Interventions
CHUM	Centre hospitalier de l’Université de Montréal
CRPS	Complex regional pain syndrome
CSF	Cerebrospinal fluid
FBSS	Failed back surgery syndrome
IPG	Implantable pulse generator
IQR	Interquartile range
MME	Morphine milligram equivalent
MRI	Magnetic resonance imaging
SCS	Spinal cord stimulation
SD	Standard deviation
STROBE	Strengthening the Reporting of Observational Studies in Epidemiology
VAS	Visual analog scale
